# Supplementary material for: Divergent aging of nulliparous and parous mammary glands reveals IL33+ hybrid epithelial cells
Source: Nat Commun. 2026 Jan 21;17:1898. doi: 10.1038/s41467-026-68611-0 (PMC12923676; doi:10.1038/s41467-026-68611-0)
Supplement: Supplementary file 1 — Supplementary Information [file 41467_2026_68611_MOESM1_ESM.pdf]

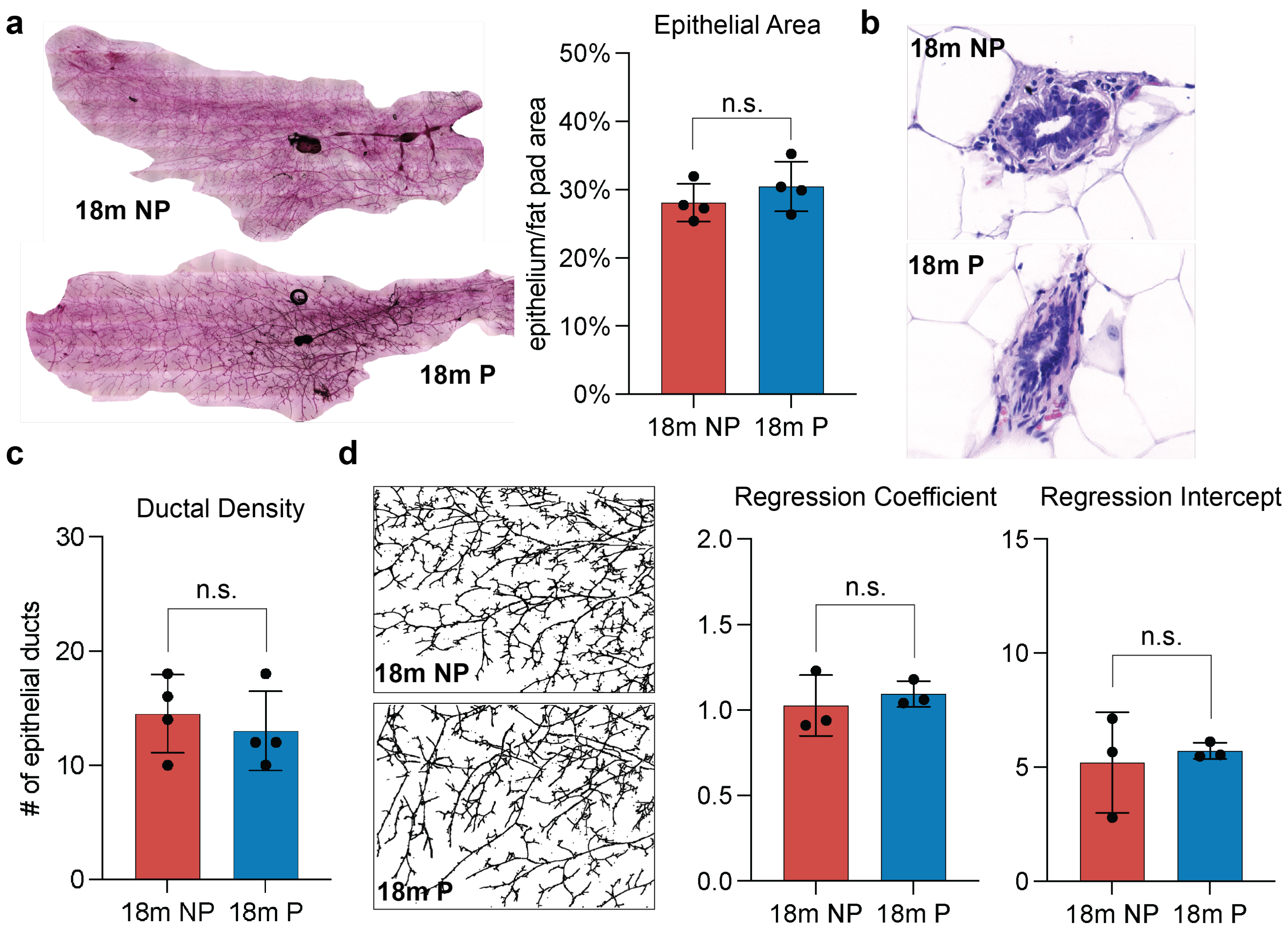

Supplementary Figure 1. Wholemount and H&E analysis of aged, parous and nulliparous mammary glands. (a) Representative wholemount images of cleared mammary glands from aged, parous and nulliparous mice stained with Carmine Alum (left) and the quantification of epithelial area from wholemount images (right). (b) Representative H&E images of epithelial ducts. (c) Quantification of ductal density per field of view from H&E images. (d) Representative images of skeletonized epithelial structures (left) used in Sholl analysis to quantify branching complexity (regression coefficient and regression intercept). Statistical significance was determined by performing unpaired Student's t-tests. n = 4 mice (a-c) or 3 mice (d). Data are presented as mean values +/- S.D. Scale bar = 1000 μm (a, d) and 100 μm (c). Source data are provided as a Source Data file.

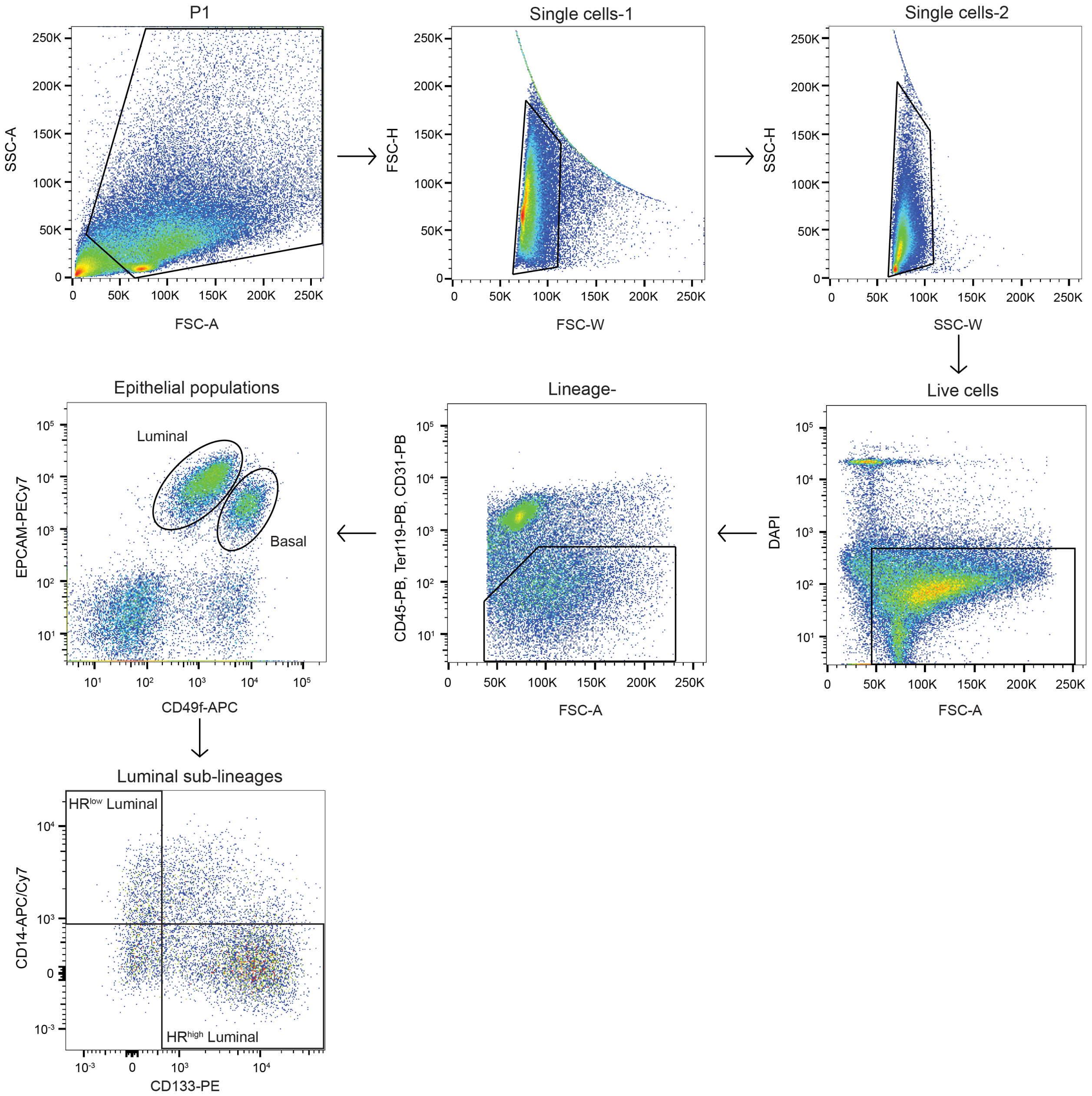

Supplementary Figure 2. Gating strategy for flow cytometry and fluorescence-activated cell sorting experiments. Single cell suspensions of mammary glands were analyzed on a BD Aria Cell Sorter. Cellular debris was removed in the "P1" SSC-A x FSC-A gate (top left). Single cells are isolated through two singlet gates using FSC-H x FSC-W and SSC-H x SSC-W (top row, middle and right panel). Live cells (DAPI-) are isolated (second row, right panel) and immune cells (CD45-Pacific Blue), red blood cells (Ter119-Pacific Blue), and endothelial cells (CD31-Pacific

Blue) are gated out through a Lin(-) gate (second row, middle panel). Basal (CD49f^{hi}/EPCAM$^{med/low}$) and luminal (CD49f^{low}/EPCAM^{hi}) epithelial cells are then isolated from the Lin(-) population (second row, left panel). Luminal cells are divided into HR^{high} and HR^{low} sublineages using CD14-APC/Cy7 and CD133-PE, respectively (third row, left panel). HR^{high} and HR^{low} luminal gates were set based on FMO negative controls.

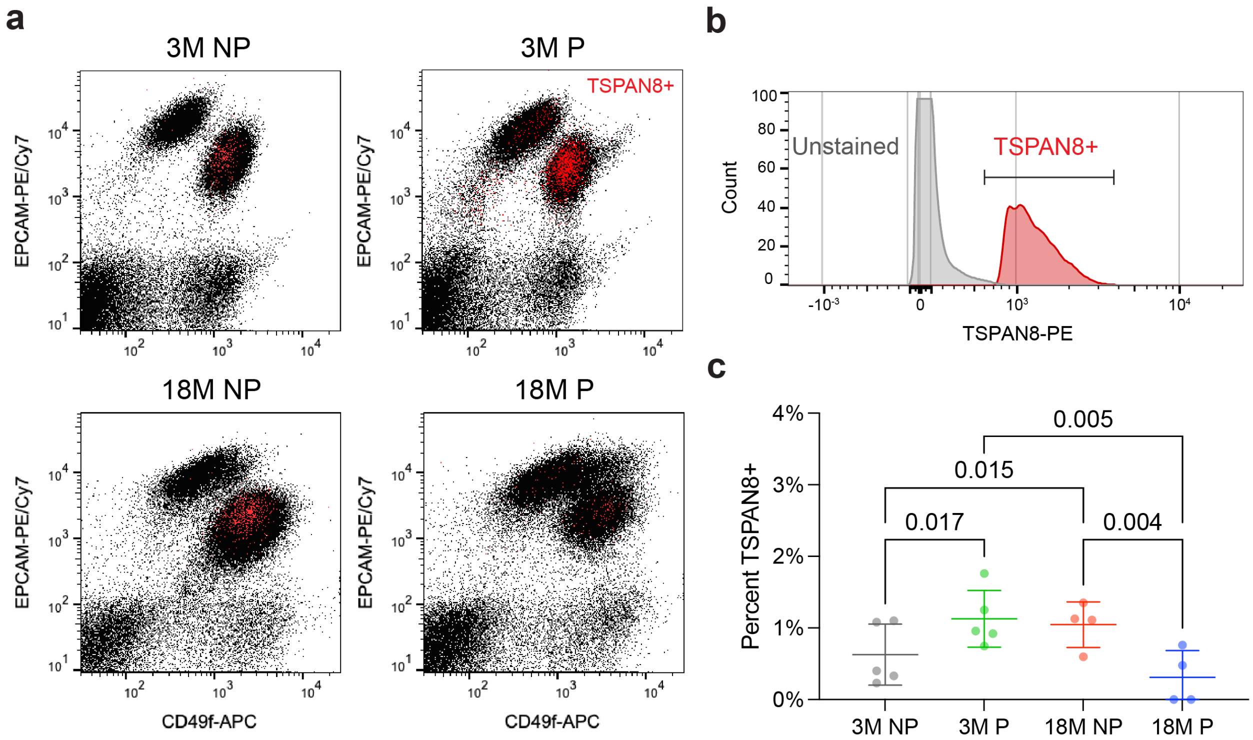

Supplementary Figure 3. TSPAN8+ MECs are enriched in young parous and aged nulliparous mammary glands. (a) Representative flow cytometry plots of DAPI-/CD45-/CD31-/Ter119- single cells from 3-month nulliparous (3M NP), 3-month parous (3M P), 18-month nulliparous (18M NP), and 18-month parous (18M P) mammary glands. TSPAN8+ MECs are displayed in red. (b) Histogram of the gating strategy for the identification of TSPAN8+ MECs. Unstained cells (negative control) are shown in gray. Compensation beads stained with a PE-conjugated TSPAN8 antibody are shown in red. (c) Percentage of TSPAN8+ MECs as a fraction of the total Lin- population across comparison groups (n = 4-5 mice per group). Statistical significance was determined by performing two-way ANOVA with Tukey's multiple comparisons test. Data are presented as mean values +/- S.D. Source data are provided as a Source Data file.

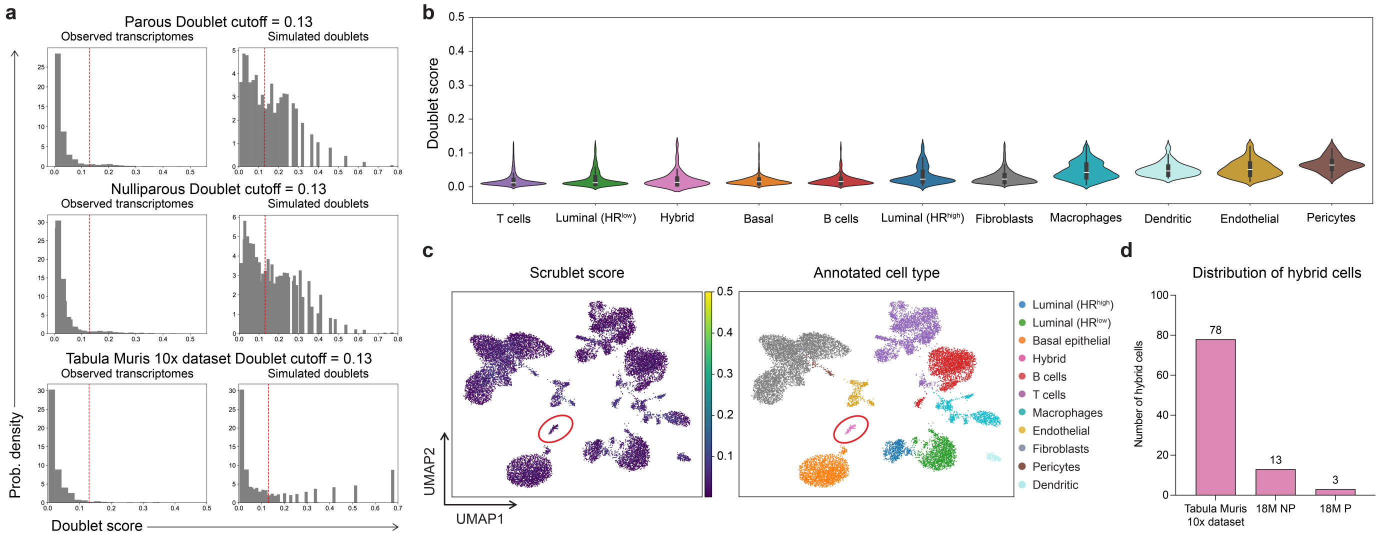

Supplementary Figure 4. Doublet detection and removal in scRNA-seq data from 18-month nulliparous and parous mammary glands. (a) Histograms of observed (left) and simulated (right) doublet scores for 18-month parous (top), 18-month nulliparous (middle), and 18-month nulliparous (bottom, Tabula Muris). Doublet scores were calculated with Scrublet and cutoff values were determined for each dataset by the minimum of the two modes in the simulated doublet histograms. (b) Doublet scores of each cell cluster after removing predicted doublets. (c) UMAP plot of scRNA-seq data from 18-month nulliparous and parous mammary glands colored by doublet score (left) and by annotated cell type (right). Red circle denotes the hybrid cell population. (d) Distribution of hybrid cell count across the three datasets. See methods for further details on the processing and quality control of scRNA-seq data. Source data are provided as a Source Data file.

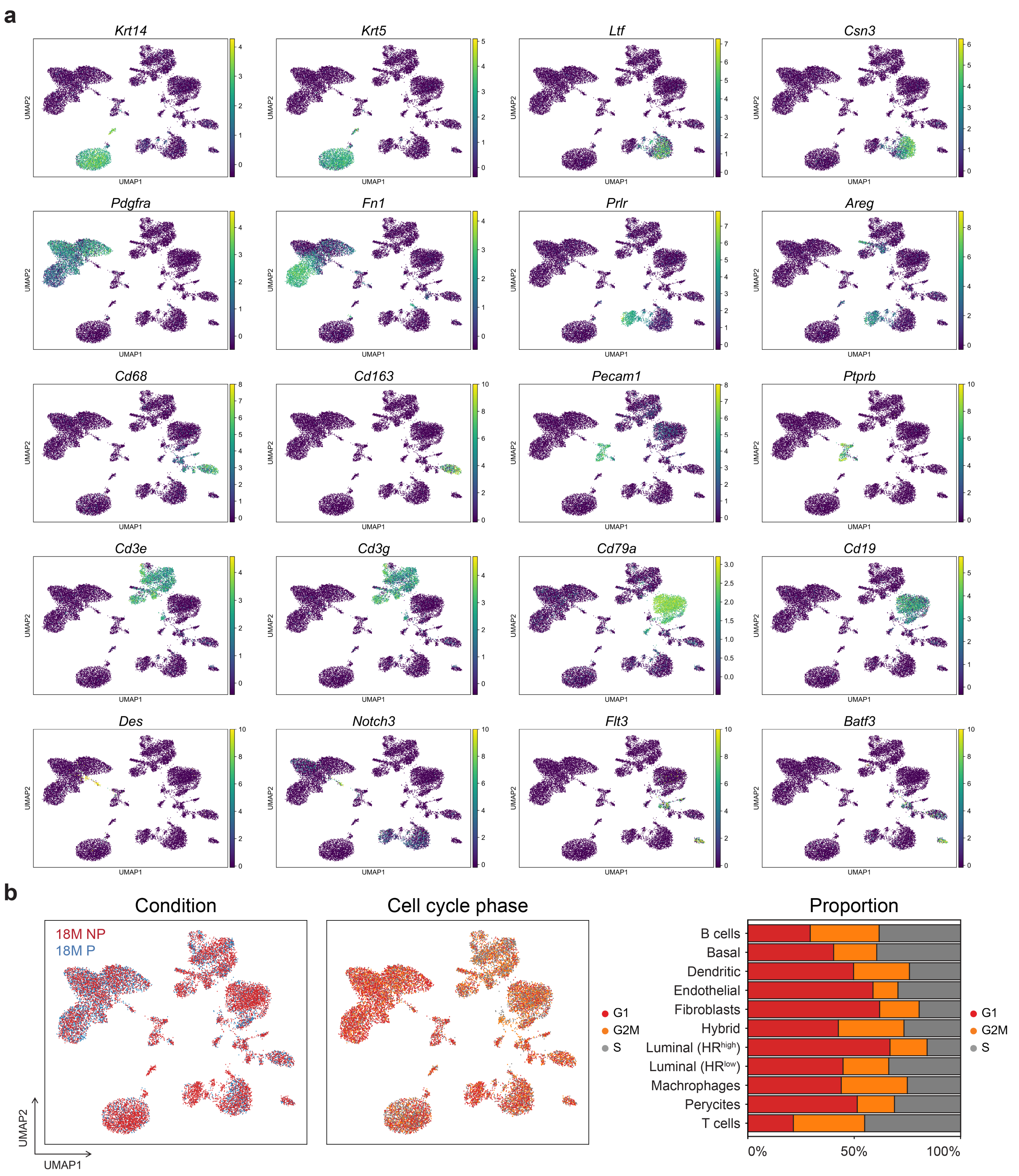

Supplementary Figure 5. Cell type annotation and cell cycle analysis of single cell RNA sequencing data. (a) UMAP plots colored by expression of lineage markers used for cell type annotation (basal, luminal-HR^{low}, luminal-HR^{high}, fibroblasts, macrophages, endothelial, T cells, B cells, pericytes, and dendritic cells). (b) UMAP plot colored by condition (18M P and 18M NP, left)

and cell cycle stage (middle). The proportion of cells in G1, G2M, and S phase are represented in a bar chart and stratified by cell type (right).

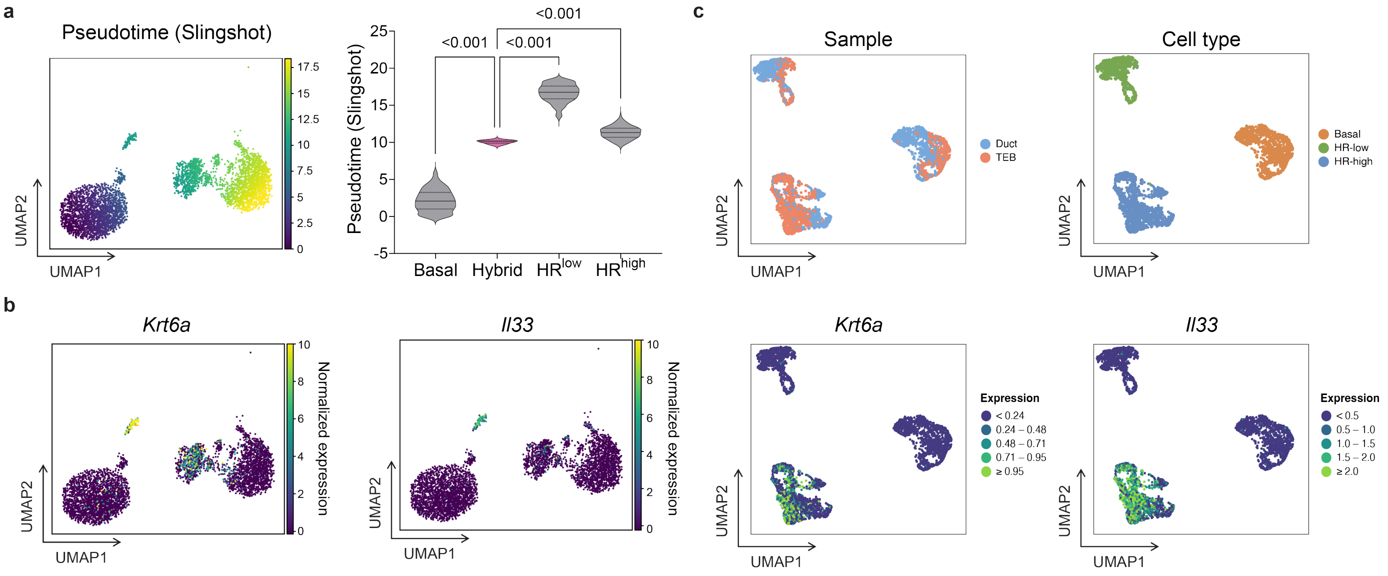

Supplementary Figure 6. *Krt6a* and *Il33* are enriched in hybrid cells and terminal end buds (TEBs). (a) Pseudotime scores inferred with Slingshot represented as a UMAP (left) and violin plot (right) across epithelial cell types from 18-month parous and nulliparous mammary glands. Data are presented as violin plots with quartiles represented by dashed lines and median values represented by solid lines. (b) UMAP plots of epithelial cells from 18-month parous and nulliparous mammary glands colored by *Krt6a* expression (left) and *Il33* expression (right)—see also Fig. 2c. (c) UMAP plots of epithelial cells from TEBs and ducts of pubertal mice (GSE164017) colored by sample (top left), annotated cell type (top right), *Krt6a* expression (bottom left), and *Il33* expression (bottom right)—see also Fig. 3h. Statistical significance was determined by performing two-way ANOVA with Tukey's multiple comparisons test. Source data are provided as a Source Data file.

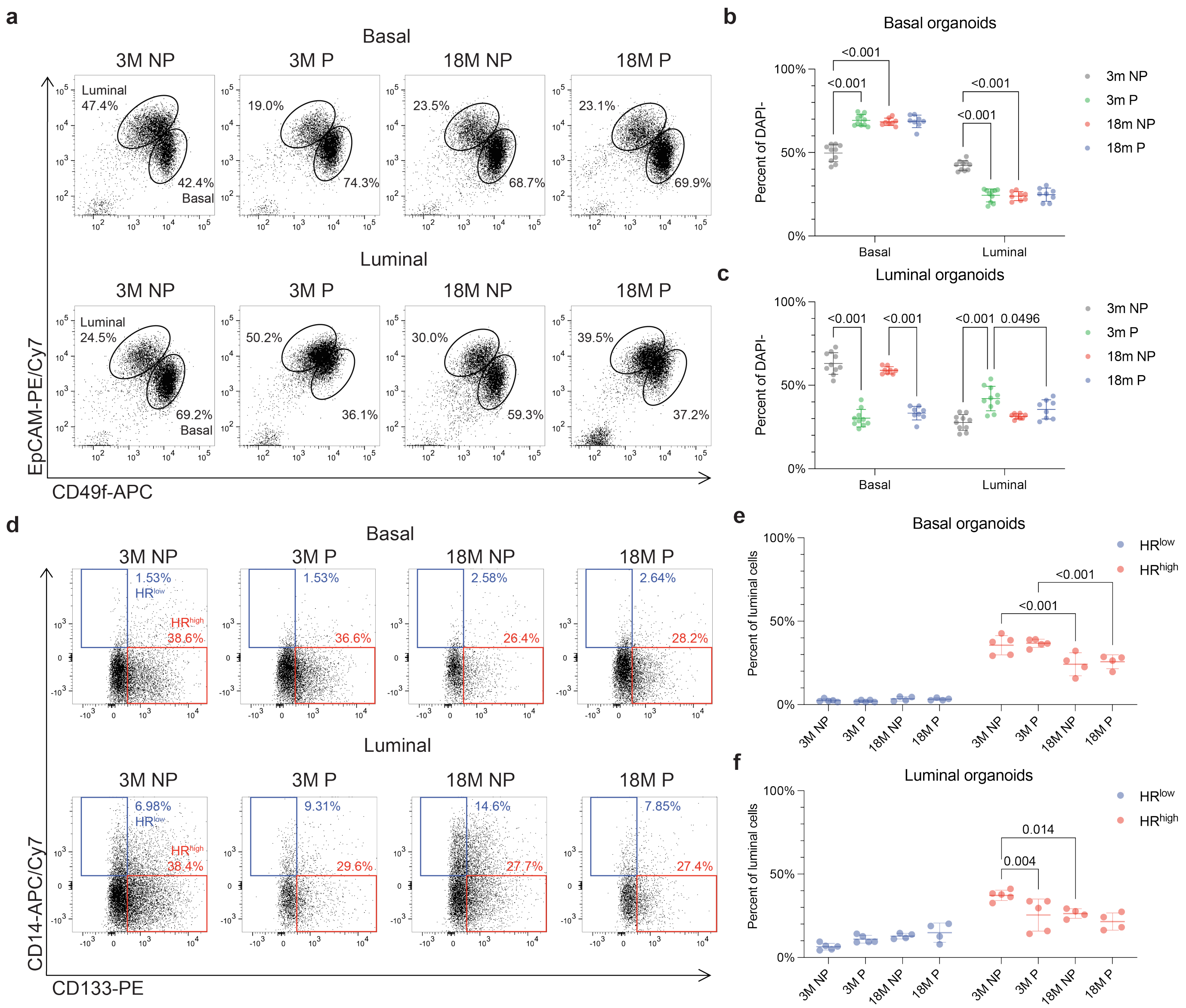

Supplementary Figure 7. Flow cytometry analysis of basal and luminal organoids derived from 3- and 18-month, parous and nulliparous mammary glands. (a) Representative flow cytometry plots of DAPI- single cells from 3-month nulliparous (3M NP), 3-month parous (3M P), 18-month nulliparous (18M NP), and 18-month parous (18M P) basal (top) and luminal (bottom) organoids. Basal (EPCAM$^{med/low}$CD49f^{high}) and luminal (EPCAM^{high}CD49f$^{med/low}$) cells are denoted by representative gates. (b) Percentage of basal and luminal cells as a fraction of DAPI- single cells in basal organoids across comparison groups. n = 4 (18M NP, 18M P) or 5 (3M NP, 3M P) mice/group, with 2 technical replicates per mouse. (c) Percentage of basal and luminal cells as a

fraction of DAPI- single cells in basal organoids across comparison groups. n = 4 (18M NP, 18M P) or 5 (3M NP, 3M P) mice/group, with 2 technical replicates per mouse. (d) Representative flow cytometry plots of luminal cells from 3M NP, 3M P, 18M NP, and 18M P basal (top) and luminal (bottom) organoids. (e, f) Quantification of HR^{low} (CD14+) and HR^{high} (CD133+) luminal cells in basal (e) and luminal (f) organoids. n = 4 (18M NP, 18M P) or 5 (3M NP, 3M P) mice/group. Statistical significance was determined by performing two-way ANOVA with Tukey's multiple comparisons test. P values are displayed for significant differences only. Source data are provided as a Source Data file.

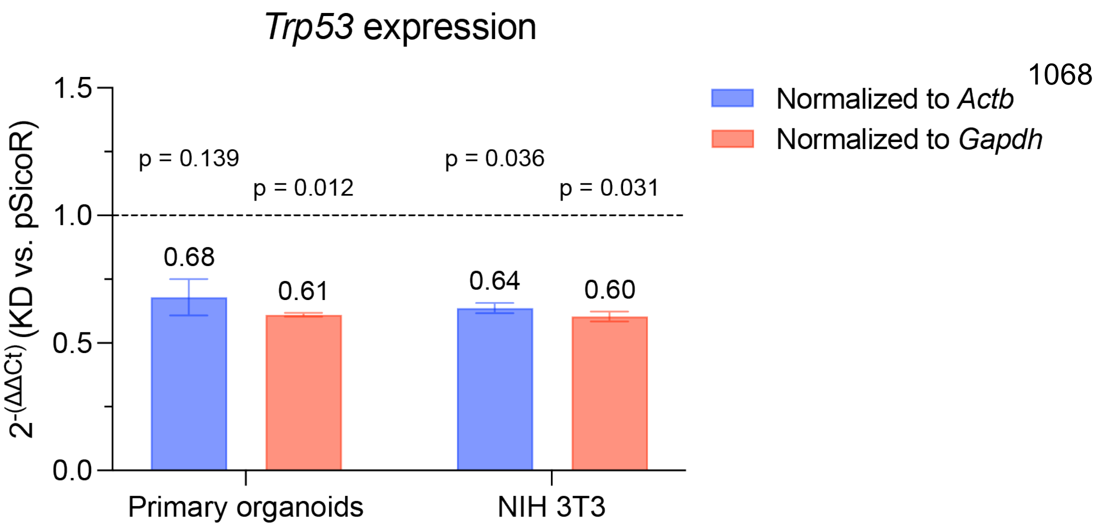

Supplementary Figure 8. Validation of short hairpin RNA-mediated *Trp53* knock-down. Expression ($2^{-\Delta\Delta Ct}$) values determined by RT-qPCR in primary mouse mammary epithelial organoids and NIH 3T3 cells infected with sh*Trp53*- or *pSicoR*-containing lentivirus. Data are normalized to the housekeeping genes *Actb* (blue) and *Gapdh* (red). Statistical significance was determined by performing one sample t-tests. n = 2 independent experiments. Data are presented as mean values +/- S.E.M. Source data are provided as a Source Data file.

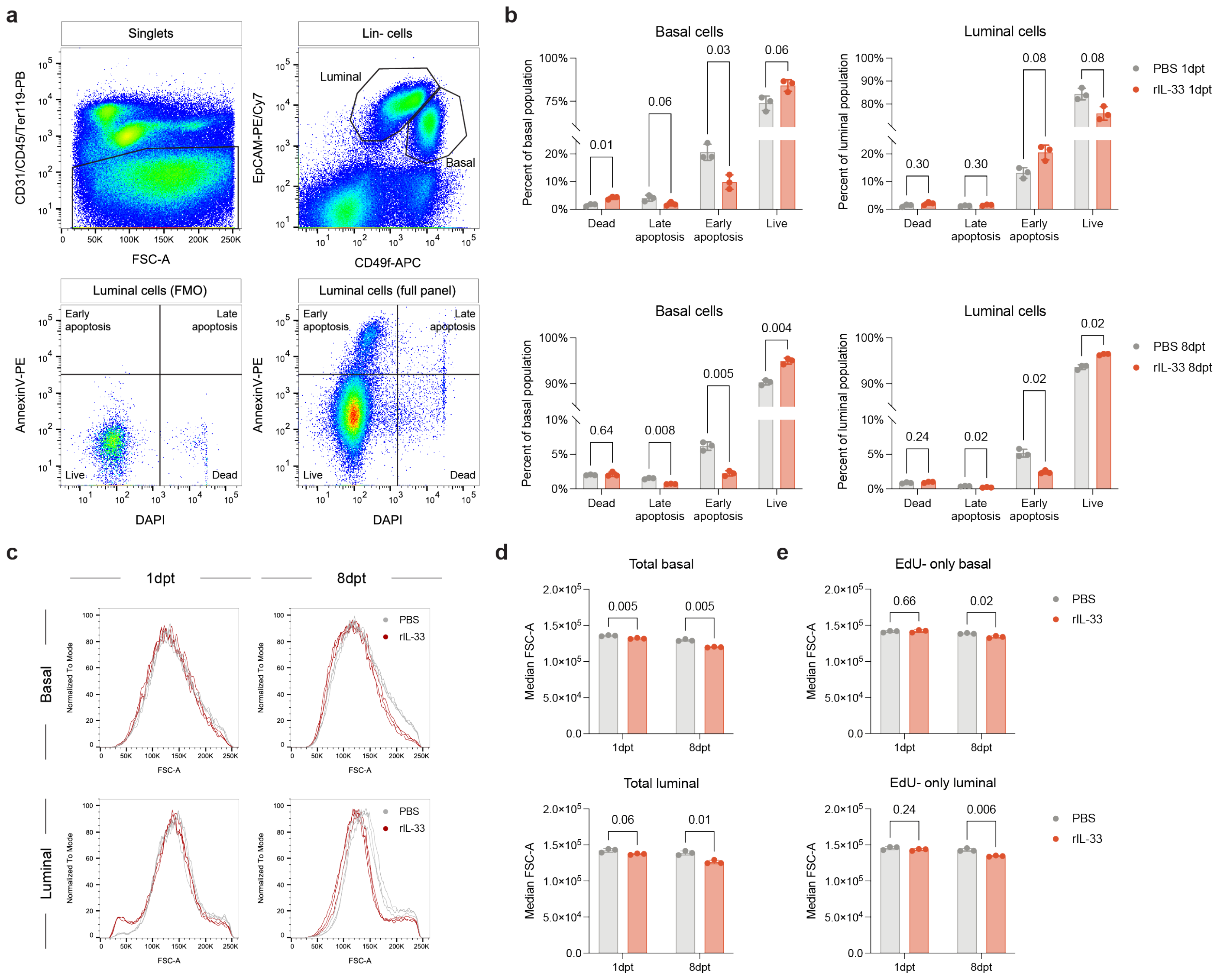

Supplementary Figure 9. Cell death flow cytometry analysis of IL33-treated mice at 1 and 8 days post treatment. (a) Representative flow plots depicting the gating strategy for luminal cell death analysis using DAPI and Annexin-V. FMO = fluorescence minus one. (b) Quantification of cell death stages for basal (left) and luminal (right) cells from mice treated with IL33 at 1dpt (top panel) and 8dpt (bottom panel). (c) Histograms of FSC-A for basal (top panel) and luminal (bottom panel) populations across treatment regimens (left to right: 1dpt, 8dpt). PBS-treated mice are represented in gray, IL33-treated mice are represented in red (d) Quantification of median FSC-A values shown in (c) for total basal (top) and total luminal (bottom) populations. (e) Quantification of median FSC-A values for EdU- basal (top) and EdU- luminal (bottom) populations. All mice of a given treatment regimen were analyzed at the same time and under identical cytometer settings.

All analyses were performed on filtered epithelial populations as shown in **Supplementary Fig. 2**. Statistical significance was determined by performing multiple unpaired t-tests with Holm-Šídák's correction (b) or two-way ANOVA with Tukey's correction (d, e). Data are presented as mean values +/- S.D. (b, d, e). Source data are provided as a Source Data file.

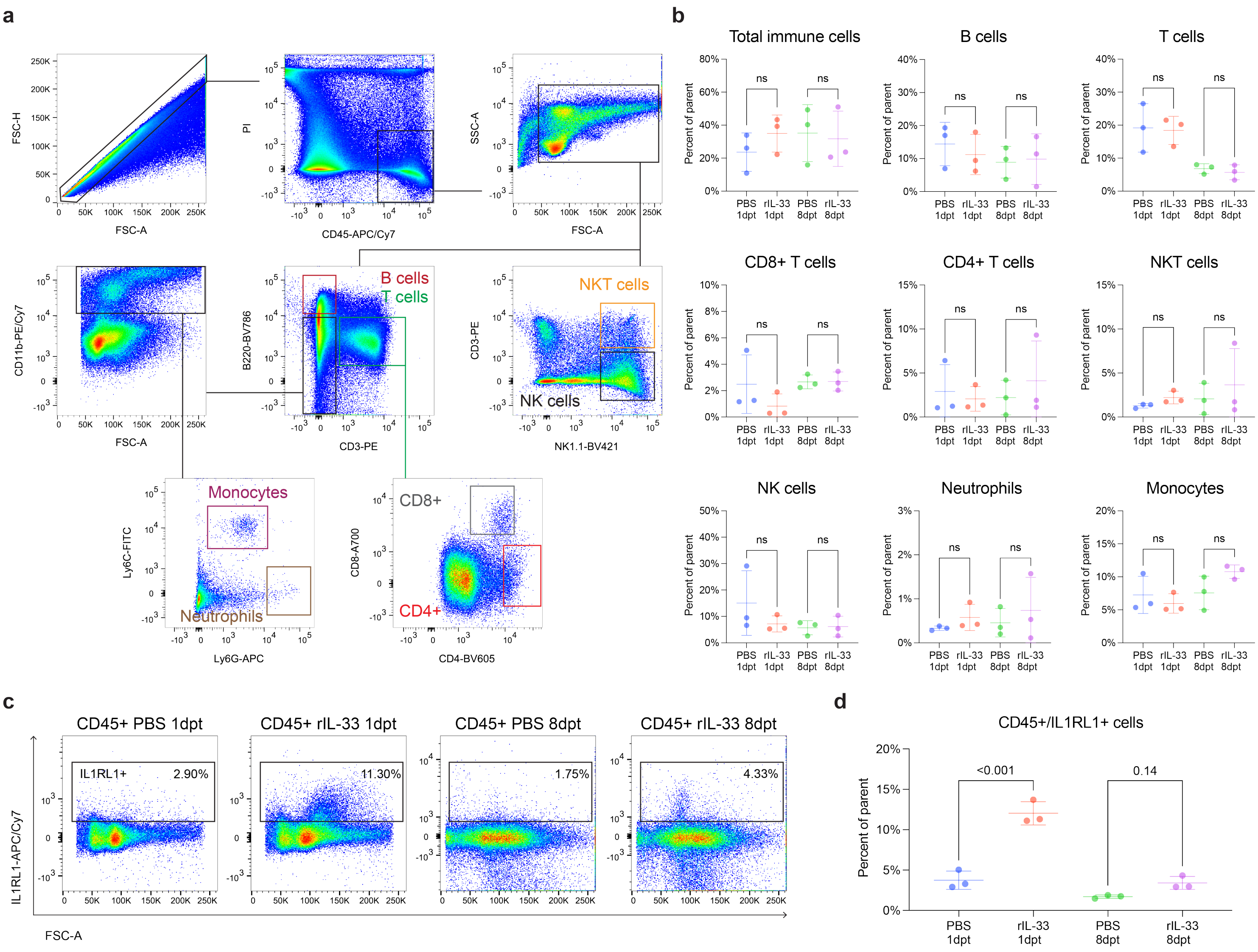

Supplementary Figure 10. Flow cytometry analysis of immune cell populations of mammary glands treated with recombinant IL-33 (rIL-33). (a) Gating strategy for the quantification of immune cell populations in mouse mammary glands exposed to rIL-33 or PBS at 1dpt and 8dpt. Single cells are isolated by FSC-H x FSC-A and live (PI-) immune (CD45+) cells are gated for analysis. Debris are removed by SSC-A x FSC-A. Remaining cells are then gated for NKT cell (CD3+/NK1.1+) NK cell (CD3-/NK1.1+), B cell (CD3-/B220+), and T cell (CD3+/B220-) populations. The T cell population is then stratified into CD4+ and CD8+ T cells. CD3-/B220-/CD11b+ cells are analyzed for monocytes (Ly6C+) and neutrophils (Ly6G+). (b) Quantification of immune cell populations (n = 3 mice). (c) Representative flow cytometry plots of PI-/CD45+ immune cells gated for IL1RL1+ cells in mouse mammary glands exposed to rIL-33 or PBS at

1dpt and 8dpt. (d) Quantification of IL1RL1+ immune cells as a fraction of total immune cells across conditions (n = 3 mice/condition). Statistical significance was determined by performing two-way ANOVA with Holm-Šídák's multiple comparisons test. Data are presented as mean values +/- S.D. Source data are provided as a Source Data file.

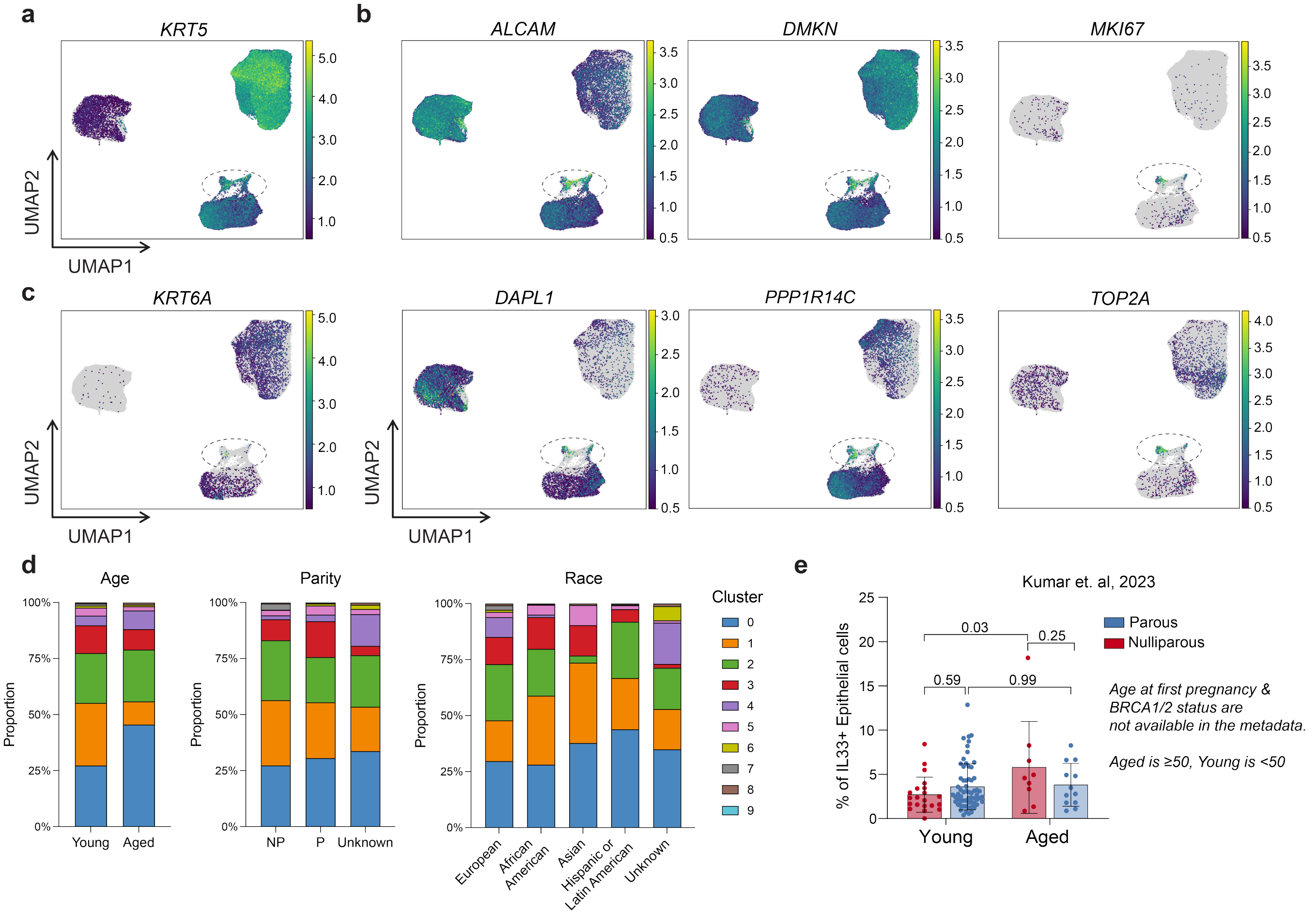

Supplementary Figure 11. Identification of hybrid MECs in the Human Breast Cell Atlas (HBCA). (a) UMAP plot of *KRT5* expression in epithelial clusters from the HBCA. Oval with dashed lines denote the hybrid cell cluster. (b) UMAP plots of hybrid cell markers (left and middle columns) and proliferation markers (right column) in epithelial clusters from the HBCA. Cells with no expression of the gene are plotted in grey for visualization. (c) UMAP plot of *KRT6A* expression in epithelial clusters from the HBCA. (d) Distribution of sub-clusters across age groups (left), parity status (middle), and race (right). (e) Percentage of IL33+ cells in the Kumar et al. dataset split by age and parity. Please note that age at first pregnancy, BRCA1/2 status are not available in the metadata. Data are presented as mean values +/- S.D. Statistical significance was determined by a one-way ANOVA. Source data are provided as a Source Data file.

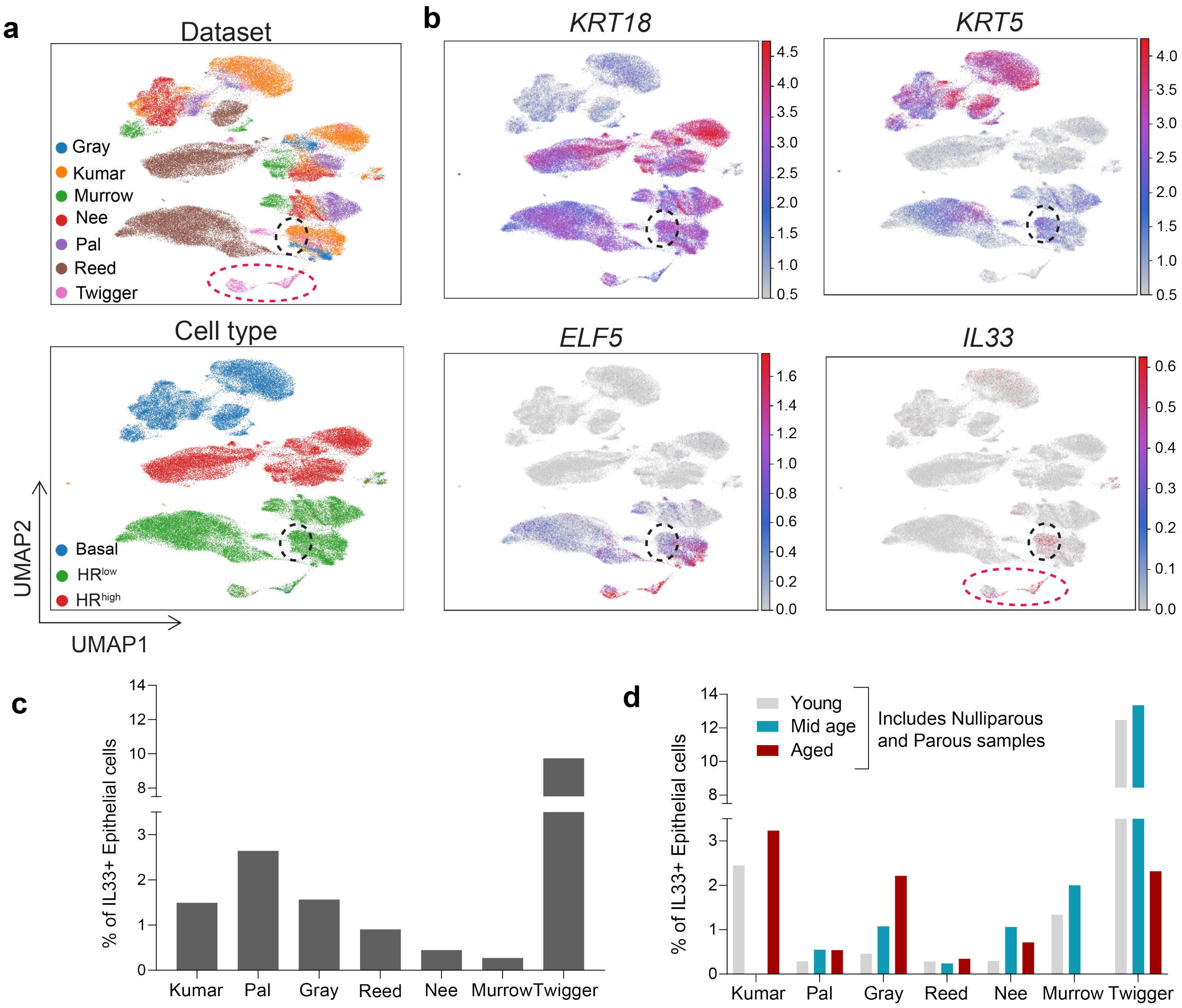

Supplementary Figure 12: Analysis of iHBCA. (a) UMAP of iHBCA annotated by dataset (left) and cell type (right). (b) Expression of KRT18, KRT5, ELF5 and IL33 in iHBCA. IL33+ cluster is highlighted with a dashed circle. (c) Percentage of IL33+ cells across all datasets. (d) Percentage of IL33+ cells in each dataset split by age. Source data are provided as a Source Data file.

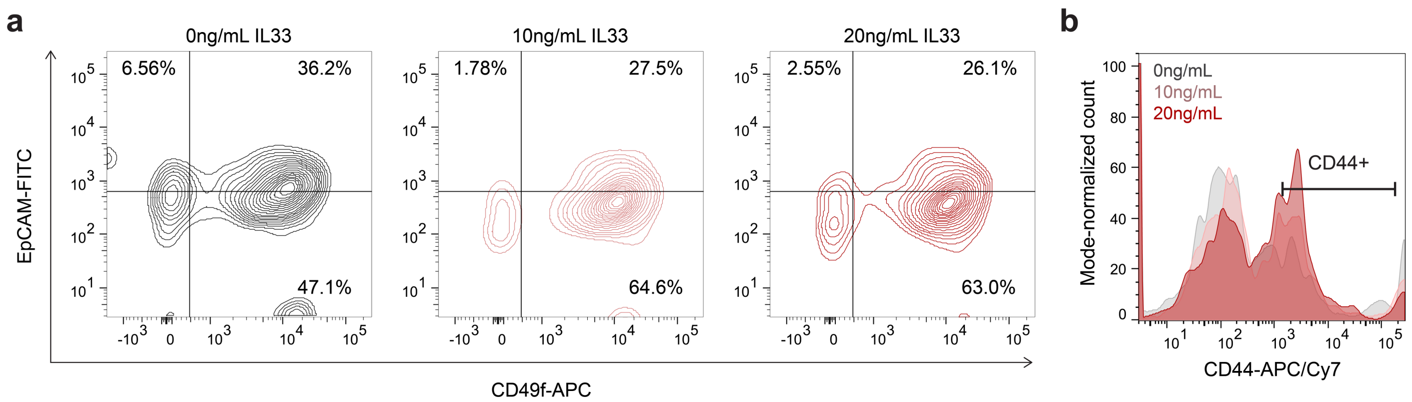

Supplementary Figure 13. Gating strategy for flow cytometry analysis of HMECs cultured in 3D. (a, b) Representative flow plots depicting the gating of EpCAM/CD49f quadrants (a) and CD44+ cells (b) in HMECs cultured in 3D.

Supplementary Tables

Supplementary Table 1. Publication data of other datasets analyzed for hybrid cells

Organism	Publication	Accession (Database)	Epithelial Cell Count
Mouse	Angarola et al., 2024	GSE216542 (GEO)	7,308
Mouse	Liang et al., 2025	GSE205573 (GEO)	22,042
Mouse	Tabula Muris Senis., 2020, Mammary Gland (Smart-seq2)	GSM2967054 (GEO)	3,282
Human	Kumar et al., 2023	GSE195665 (GEO)	240,804
Human	Reed et al., 2024	E-MTAB-13664 (ArrayExpress)	352,496

Supplementary Table 2. Antibodies and viability dyes

**Antibody**	**Dilution**	**Clone**	**Manufacturer**	**Catalog Number**
PE/Cyanine7 anti-mouse CD326 (EPCAM)	1:200	G8.8	BioLegend	118216
APC anti-human/mouse CD49f	1:100	GoH3	BioLegend	313616
PE anti-mouse CD133	1:100	315-2C11	BioLegend	141203
APC/Cyanine7 anti-mouse CD14	1:50	Sa14-2	BioLegend	123318
PE anti-mouse TSPAN8	1:200	FAB6524P	R&D Systems	657909
APC/Fire™ 750 anti-mouse IL-33Rα (IL1RL1, ST2)	1:100	DIH9	BioLegend	145326

Pacific Blue™ anti-mouse TER-119/Erythroid	1:200	TER-119	BioLegend	116232
Pacific Blue™ anti-mouse CD31	1:200	390	BioLegend	102422
Pacific Blue™ anti-mouse CD45	1:200	S18009F	BioLegend	102422
APC/Cyanine7 anti-mouse CD45	1:400	QA17A26	BioLegend	157617
PE anti-mouse CD3	1:200	17A2	BioLegend	100205
Brilliant Violet 605™ anti-mouse CD4	1:400	RM4-5	BioLegend	100547
Alexa Fluor® 700 anti-mouse CD8a	1:400	53-6.7	BioLegend	100729
Brilliant Violet 785™ anti-mouse/human CD45R/B220	1:200	RA3-6B2	BioLegend	103245
PE/Cyanine7 anti-mouse CD11b	1:800	M1/70	BioLegend	101215
Alexa Fluor® 488 anti-mouse Ly-6C	1:400	HK1.4	BioLegend	128021
APC anti-mouse Ly-6G	1:400	1A8	BioLegend	127613
Pacific Blue™ anti-mouse NK1.1	1:100	PK136	BioLegend	108721
PE Annexin V	1:20	-	Biolegend	640908
PE/Cyanine7 anti-human/mouse CD49f	1:200	GoH3	BioLegend	313621
APC/Cyanine7 anti-mouse/human CD44	1:100	IM7	BioLegend	103027

FITC anti-human CD326 (EpCAM)	1:200	CO-171A	BioLegend	369813
Rabbit anti-human CSN1S1	1:200	-	Novus Biologicals	NBP2-55090
Rabbit anti-mouse KRT6A	1:100	-	BioLegend	905701
Chicken anti-mouse KRT5	1:100	-	BioLegend	905901
Goat anti-mouse alpha smooth muscle actin	1:200	-	Abcam	ab21027
Rat anti-mouse KRT8	1:200	TROMA-1	Developmental Studies Hybridoma Bank	NA
Chicken anti-mouse IL33	1:100	-	Invitrogen	PIPA547007
Donkey anti-goat Alexa Fluor 488	1:400	-	Invitrogen	A11034
Donkey anti-chicken Alexa Fluor 488	1:400	-	Jackson ImmunoResearch Laboratories	703-005-155
Donkey anti-rabbit Alexa Fluor 594	1:400	-	Jackson ImmunoResearch Laboratories	711-585-152
Donkey anti-rat Alexa Fluor 647	1:400	-	Jackson ImmunoResearch Laboratories	712-605-150
4',6-DIamidino 2 phenylidole, Dihydrochloride (DAPI)	1:1000 (IF) or 1:10,000 (flow)	-	Life Technologies	D1306
Zombie UV™ Fixable Viability Kit	1:500	-	Biolegend	423107
