# Supplementary material for: Divergent aging of nulliparous and parous mammary glands reveals IL33+ hybrid epithelial cells
Source: Nat Commun. 2026 Jan 21;17:1898. doi: 10.1038/s41467-026-68611-0 (PMC12923676; doi:10.1038/s41467-026-68611-0)
Supplement: Supplementary file 2 — Reporting Summary [file 41467_2026_68611_MOESM2_ESM.pdf]

nature portfolio

Corresponding author(s): Shaheen Sikandar

Last updated by author(s): 12/15/2025

Reporting Summary

Nature Portfolio wishes to improve the reproducibility of the work that we publish. This form provides structure for consistency and transparency in reporting. For further information on Nature Portfolio policies, see our Editorial Policies and the Editorial Policy Checklist.

Statistics

For all statistical analyses, confirm that the following items are present in the figure legend, table legend, main text, or Methods section.

n/a	Confirmed	
☐	☒	The exact sample size (*n*) for each experimental group/condition, given as a discrete number and unit of measurement
☐	☒	A statement on whether measurements were taken from distinct samples or whether the same sample was measured repeatedly
☐	☒	The statistical test(s) used AND whether they are one- or two-sided<br>*Only common tests should be described solely by name; describe more complex techniques in the Methods section.*
☒	☐	A description of all covariates tested
☐	☒	A description of any assumptions or corrections, such as tests of normality and adjustment for multiple comparisons
☐	☒	A full description of the statistical parameters including central tendency (e.g. means) or other basic estimates (e.g. regression coefficient) AND variation (e.g. standard deviation) or associated estimates of uncertainty (e.g. confidence intervals)
☐	☒	For null hypothesis testing, the test statistic (e.g. *F*, *t*, *r*) with confidence intervals, effect sizes, degrees of freedom and *P* value noted<br>*Give P values as exact values whenever suitable.*
☒	☐	For Bayesian analysis, information on the choice of priors and Markov chain Monte Carlo settings
☒	☐	For hierarchical and complex designs, identification of the appropriate level for tests and full reporting of outcomes
☒	☐	Estimates of effect sizes (e.g. Cohen's *d*, Pearson's *r*), indicating how they were calculated

Our web collection on statistics for biologists contains articles on many of the points above.

Software and code

Policy information about availability of computer code

Data collection	Flowcytometry analysis and cell sorting was performed using the BD FACSAria instrument. Microscopy data was collected using Zeiss Live Cell microscope, Leica Widefield microscope and Solamere Spinning Disk Confocal as described in the methods section. Sequencing was performed on an Illumina NovaSeq 6000
Data analysis	Data analysis was performed using Flowjo (10.10.0), BioDoc.AI, ImageJ/Fiji, 10x Genomics Cell Ranger pipeline, Scrublet, Scanpy (v1.11.0), Harmony (v0.0.4), niform Manifold Approximation and Projection (sc.tl.umap), PAGA (v1.2), pyslingshot (v0.1.3), CytoTRACE 2 (v1.0.0) in Python. Visualizations were generated with Scanpy, Seaborn, and Matplotlib. Statistical tests were implemented using scipy.stats.mannwhitneyu (SciPy v1.11.1), multipletests from statsmodels.stats.multitest or Graphpad Prism (10.2.2)

For manuscripts utilizing custom algorithms or software that are central to the research but not yet described in published literature, software must be made available to editors and reviewers. We strongly encourage code deposition in a community repository (e.g. GitHub). See the Nature Portfolio guidelines for submitting code & software for further information.

Data

Policy information about availability of data

All manuscripts must include a data availability statement. This statement should provide the following information, where applicable:

- Accession codes, unique identifiers, or web links for publicly available datasets
- A description of any restrictions on data availability
- For clinical datasets or third party data, please ensure that the statement adheres to our policy

Data generated or analyzed during this study are included in this published article (and its supplemental information files). Data needed to evaluate the conclusions in the paper are present in the paper and/or the Supplemental Materials. Source data files are included in the manuscript Single-cell and bulk RNA-sequencing data generated in this study have been deposited in the Gene Expression Omnibus with the primary accession code GSE272932. All bioinformatics tools used in this study are published and publicly available.

Research involving human participants, their data, or biological material

Policy information about studies with human participants or human data. See also policy information about sex, gender (identity/presentation), and sexual orientation and race, ethnicity and racism.

Reporting on sex and gender	NA - only publicly available datasets were used that already specify the sex and gender
Reporting on race, ethnicity, or other socially relevant groupings	NA - only publicly available datasets were used that already specify the race, ethnicity or other socially relevant groupings.
Population characteristics	NA - only publicly available datasets were used that already specify the population characteristics.
Recruitment	NA
Ethics oversight	NA

Note that full information on the approval of the study protocol must also be provided in the manuscript.

Field-specific reporting

Please select the one below that is the best fit for your research. If you are not sure, read the appropriate sections before making your selection.

☒ Life sciences ☐ Behavioural & social sciences ☐ Ecological, evolutionary & environmental sciences

For a reference copy of the document with all sections, see nature.com/documents/nr-reporting-summary-flat.pdf

Life sciences study design

All studies must disclose on these points even when the disclosure is negative.

Sample size	sample size was not predetermined to ensure adequate power to detect a prespecified effect size. Sample size was limited based on approved animal numbers. For in vitro experiments, no statistical method was used to predetermine sample size; all samples were analyzed equally, and randomization was not required. Sample size for in vitro experiments were conducted at n =/> 3 according to historical experience with similar type of experiments performed in our laboratory. This sample size typically yields standard error <25% of the mean value. For in vivo experiments, no statistical test was used to predetermine sample size; group sizes were informed by pilot and prior studies. We used at least 5 mice per group which is sufficient to detect meaningful biological difference.
Data exclusions	Cells in scRNA Seq exhibiting high mitochondrial and ribosomal gene content were excluded. No other data were excluded from the analysis.
Replication	All experiments were performed in 3-5 biological replicates or more as indicated in the manuscript. All biological and technical replicates are included in the manuscript. All replication attempts were successful - no data are excluded from the analysis.
Randomization	Mice and/or cells were randomized for treatment with IL33 and analysis on flowcytometry
Blinding	Researchers were not blinded to the study as treatment conditions or groups required knowledge of which mice/organoids should receive IL33 or the age of mice for analysis.

Reporting for specific materials, systems and methods

We require information from authors about some types of materials, experimental systems and methods used in many studies. Here, indicate whether each material, system or method listed is relevant to your study. If you are not sure if a list item applies to your research, read the appropriate section before selecting a response.

Materials & experimental systems

n/a	Involved in the study
☐	☒ Antibodies
☐	☒ Eukaryotic cell lines
☒	☐ Palaeontology and archaeology
☐	☒ Animals and other organisms
☒	☐ Clinical data
☒	☐ Dual use research of concern
☒	☐ Plants

Methods

n/a	Involved in the study
☒	☐ ChIP-seq
☐	☒ Flow cytometry
☒	☐ MRI-based neuroimaging

Antibodies

Antibodies used	All antibodies are listed in Table 2 provided in the manuscript
Validation	All antibodies are commonly used across the literature and have been validated by the manufacturer for the corresponding applications used here. All antibodies used here have been cited in more than 5 peer-reviewed articles per antibody. Whenever possible antibodies were also validated using a negative control that either lacks expression, a positive control that has expression or an IgG conjugated flourophore that does not bind to the same anigen. Antibody validation statements for each antibody are provided on the manufacturer's website for all the antibodies listed in Table 2. No antibodies were generated for this study.

Eukaryotic cell lines

Policy information about cell lines and Sex and Gender in Research

Cell line source(s)	Human primary mammary epithelial cells were purchased from ATCC (PCS-600-010)
Authentication	HMEC cells were not authenticated after purchase from ATCC
Mycoplasma contamination	Cells are routinely checked for mycoplasma contamination using PCR. HMEC bought from ATCC were negative for mycoplasma contanmination
Commonly misidentified lines (See ICLAC register)	No ICLAC cell lines were used.

Animals and other research organisms

Policy information about studies involving animals; ARRIVE guidelines recommended for reporting animal research, and Sex and Gender in Research

Laboratory animals	All mice used for this study were maintained at the UCSC Animal Facility/Vivarium in accordance with the guidelines of the Institutional Animal Care and Use Committee (Protocol #Sikas2311dn).<br>Wild-type C57BL/6 mice (young), retired breeders and aged matched mice when appropriate were purchased from Charles River Laboratories and The Jackson Laboratory. Simultaneously an aging colony was maintained in house. Only female mice were used in the study as male mice do not undergo pregnancy. All mice were housed in climate-controlled rooms with a minimum air exchange rate of eight times per hour. The ambient temperature was maintained between 20–24 °C and the relative humidity ranged from 45–65%. An automated 12:12 h light-dark cycle was implemented in all animal holding rooms. The mice were recorded for estrous and 18M mice were found to be non-cycling. Mice are euthanized by CO2 followed by cervical dislocation.
Wild animals	NA
Reporting on sex	Only female mice are used in the study as the study is focussed on understanding the impact of pregnancy on the aging of mammary stem/progenitor cells.
Field-collected samples	NA
Ethics oversight	All mice used for this study were maintained at the UCSC Animal Facility/Vivarium in accordance with the guidelines of the Institutional Animal Care and Use Committee (Protocol #Sikas2311dn).

Note that full information on the approval of the study protocol must also be provided in the manuscript.

Plants

Seed stocks	NA
Novel plant genotypes	NA
Authentication	NA

Flow Cytometry

Plots

Confirm that:

- [x] The axis labels state the marker and fluorochrome used (e.g. CD4-FITC).
- [x] The axis scales are clearly visible. Include numbers along axes only for bottom left plot of group (a 'group' is an analysis of identical markers).
- [x] All plots are contour plots with outliers or pseudocolor plots.
- [x] A numerical value for number of cells or percentage (with statistics) is provided.

Methodology

Sample preparation	L2-5 and R2-5 mammary glands were harvested, minced, and chemically digested overnight in Advanced DMEM F/12 with 1% PSA, gentle collagenase/hyaluronidase, and DNAse I at 37C, 5% CO2, and added humidity as previously described72. Briefly, partially digested glands were then mechanically digested by pipetting with a serological pipette until no tissue pieces were visible. Digested glands were washed with staining buffer (Hank's Balanced Salt Solution, 2% Bovine Calf Serum, 1% PSA) and centrifuged (1500RPM) at 4C for 5 minutes. Red blood cells were lysed with 5mL of ACK Lysis buffer for 5 minutes, and cells were washed with 15mL of staining buffer. Cells were treated with 0.25% Trypsin with EDTA and gently pipetted continuously for 2-3 minutes to digest the basement membrane. Cells were then treated with DNAse I and Dispase and pipetted continuously for 2-3 minutes to prevent clumping. The single-cell suspension was then filtered through a 40um mesh strainer and pelleted via centrifugation (1500RPM, 4C, 5 minutes). Cells were then resuspended in staining buffer and transferred to FACS tubes for staining.
Instrument	BD FACS Aria
Software	FlowJo software (10.10.0)
Cell population abundance	Cells were sorted based on purity. Post-sort purity for organoid cultures was not determined.
Gating strategy	Relevant gating strategies were determined using a FMO control for each antibody used.

- [x] Tick this box to confirm that a figure exemplifying the gating strategy is provided in the Supplementary Information.
